# Characterization of a glucose-tolerant β-glucosidase from *Anoxybacillus* sp. DT3-1

**DOI:** 10.1186/s13068-016-0587-x

**Published:** 2016-08-22

**Authors:** Chia Sing Chan, Lee Li Sin, Kok-Gan Chan, Mohd Shahir Shamsir, Fazilah Abd Manan, Rajesh Kumar Sani, Kian Mau Goh

**Affiliations:** 1 Faculty of Biosciences and Medical Engineering, Universiti Teknologi Malaysia, 81300 Skudai, Johor Malaysia; 2 Division of Genetics and Molecular Biology, Institute of Biological Sciences, Faculty of Science, University of Malaya, Kuala Lumpur, Malaysia; 3 Department of Chemical and Biological Engineering, South Dakota School of Mines and Technology, Rapid City, USA

**Keywords:** *Anoxybacillus*, Biofuel, Biomass conversion, Glucose tolerance, Glycosyl hydrolase

## Abstract

**Background:**

In general, biofuel production involves biomass pretreatment and enzymatic saccharification, followed by the subsequent sugar conversion to biofuel via fermentation. The crucial step in the production of biofuel from biomass is the enzymatic saccharification. Many of the commercial cellulase enzyme cocktails, such as Spezyme^®^ CP (Genencor), Acellerase™ 1000 (Genencor), and Celluclast^®^ 1.5L (Novozymes), are ineffectively to release free glucose from the pretreated biomass without additional β-glucosidase.

**Results:**

In this study, for the first time, a β-glucosidase DT-Bgl gene (1359 bp) was identified in the genome of *Anoxybacillus* sp. DT3-1, and cloned and heterologously expressed in *Escherichia coli* BL21. Phylogenetic analysis indicated that DT-Bgl belonged to glycosyl hydrolase (GH) family 1. The recombinant DT-Bgl was highly active on cello-oligosaccharides and *p*-nitrophenyl-β-d-glucopyranoside (*p*NPG). The DT-Bgl was purified using an Ni–NTA column, with molecular mass of 53 kDa using an SDS-PAGE analysis. It exhibited optimum activity at 70 °C and pH 8.5, and did not require any tested co-factors for activation. The *K*_m_ and *V*_max_ values for DT-Bgl were 0.22 mM and 923.7 U/mg, respectively, with *p*NPG as substrate. The DT-Bgl displayed high glucose tolerance, and retained 93 % activity in the presence of 10 M glucose.

**Conclusions:**

*Anoxybacillus* DT-Bgl is a novel thermostable β-glucosidase with low glucose inhibition, and converts long-chain cellodextrins to cellobiose, and further hydrolyse cellobiose to glucose. Results suggest that DT-Bgl could be useful in the development of a bioprocess for the efficient saccharification of lignocellulosic biomass.

## Background

Lignocellulosic agricultural and forestry wastes are regarded as abundant renewable resources and considered ideal feedstocks for biofuel production. Crop residues are produced worldwide at about 2802 × 10^6^ mega grams/year (Mg/year) for cereal crops, 3107 × 10^6^ Mg/year for 17 cereals and legumes, and 3758 × 10^6^ for 27 food crops [1]. Lignocellulosic wastes are mainly composed of cellulose, hemicellulose, and lignin. Typically, cellulose and hemicellulose comprise up to two-thirds of lignocellulosic wastes. Glycoside hydrolases (GHs) hydrolyse cellulosic and hemicellulosic fractions of plant biomass, while auxiliary activities (AA) involves in plant cell wall degradation. Both GH and AA families have been divided into 135 and 13 groups, respectively, and classified by substrate specificity or sequence identity [2]. Enzymes that deconstruct cellulose and cellobiose are indexed in GH1, GH3, GH5, GH8, GH9, GH44, GH45, AA3_1, AA9, and AA10 [3, 4]. Hemicellulose, of which the main subunits are xylan, mannan, and galactan, is structurally more complex than cellulose. Enzymes categorised as GH3, GH10, GH11, GH39, GH43, and GH52 act on xylan or xylobiose to yield their monomers [3]. Enzymes belonging to GH26 and GH38 specifically hydrolyse dimers or longer mannan chains to mannose units. Monomeric galactose is generated by the activities of GH2, GH16, GH35, GH41, and GH53 enzymes acting on the galactan polymer [3]. While most members of auxiliary group act on lignin which holds hemicellulose and cellulose together [4], AA3_1 (cellobiose dehydrogenase), AA9 (lytic polysaccharide monooxygenase, previously known as GH61), and AA10 (lytic polysaccharide monooxygenase, formerly those enzymes with carbohydrate-binding module family CBM33) are able to act on polysaccharides by oxidative reactions [5–10]. To enhance the efficiency of biomass deconstruction, the presence of AA (e.g., laccase, manganese peroxidase, cellobiose dehydrogenases, and alcohol oxidases) is important.

Cellulose from forestry and agricultural wastes can be degraded to cellodextrin and cellobiose or glucose. This last step in cellulose hydrolysis involves the use of β-glucosidase (BGL) [11]. BGL is known as a bottleneck factor in the cellulose hydrolysis process. Most commercial fungal BGLs have low stability at higher temperatures (>50 °C). In addition, glucose inhibition is a common problem for several BGL enzymes [12]. To maximize the overall biofuel production efficiency, harnessing thermostable and active BGL with high glucose tolerance is needed [13].

Currently, five complete genomes of *Anoxybacillus* spp. are available. Genome description for *Anoxybacillus flavithermus* WK1 (CP000922.1) and *Anoxybacillus gonensis* G2^T^ (CP012152.1) has been reported [14, 15], while genome data for *Anoxybacillus amylolyticus* DSM 15939^T^ (chromosome CP015438.1, plasmid pDSM15939_1 CP015439.1, and plasmid pDSM15939_2 CP015440.1), *Anoxybacillus* sp. B2M1 (CP015435.1), and *Anoxybacillus* sp. B7M1 (chromosome CP015436.1 and plasmid CP015437.1) are available in NCBI database. The genome of *A. flavithermus* WK1 contains nine genes encoding for GH13 enzymes, two genes for GH32, and one gene for each of the families GH1, GH23, GH31, GH43, GH51, and GH65 [15]. Biochemical and bioinformatics analyses have revealed that enzymes from *Anoxybacillus* spp. are important for starch industrial processes [16] and biomass deconstruction [17].

*Anoxybacillus* sp. DT3-1 (DSM 28778) is a thermophilic bacterium which was isolated from a Malaysian hot spring of a temperature of 75 °C [18], where its genome has been sequenced. A putative BGL (GH1) identified (UniProtKB M5QUM2) in *Anoxybacillus* sp. DT3-1 as well as its homologues annotated in other genomes of *Anoxybacillus* spp. have not been biochemically investigated. The purpose of this work was to characterize DT-Bgl from *Anoxybacillus* sp. DT3-1 and to evaluate its potential use in the conversion of cello-oligosaccharides to glucose.

## Results and discussion

### Protein sequence analysis

**Fig. 1:**
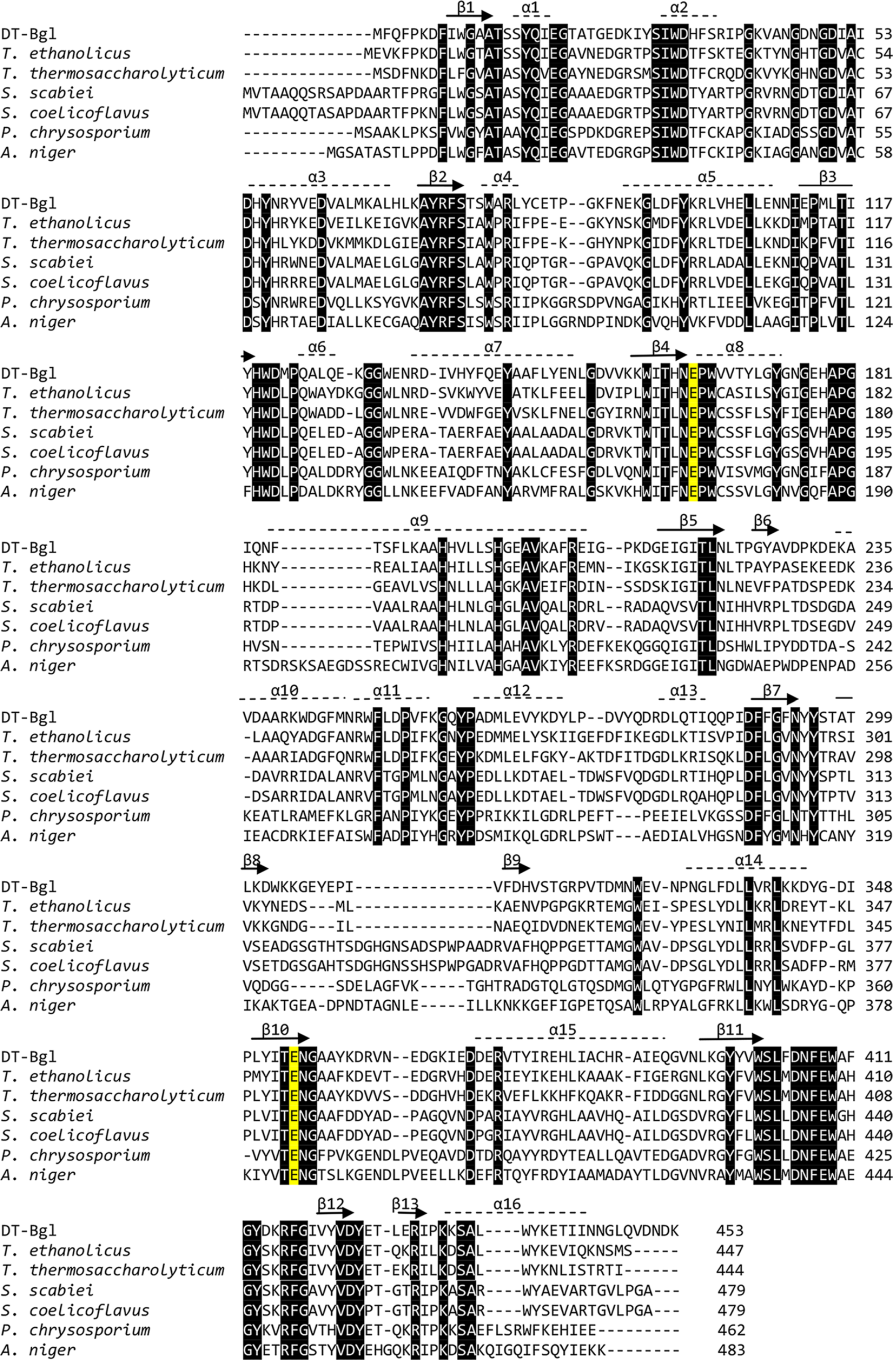
Multiple amino acid sequence alignment of DT-Bgl and GH1 BGL enzymes. The numbers flanking the sequences represent amino acid positions of each sequence, and the *black*-highlighted areas are associated with high similarity conserved regions. The two putative catalytic residues are indicated as *yellow* highlights in the alignment

**Fig. 2:**
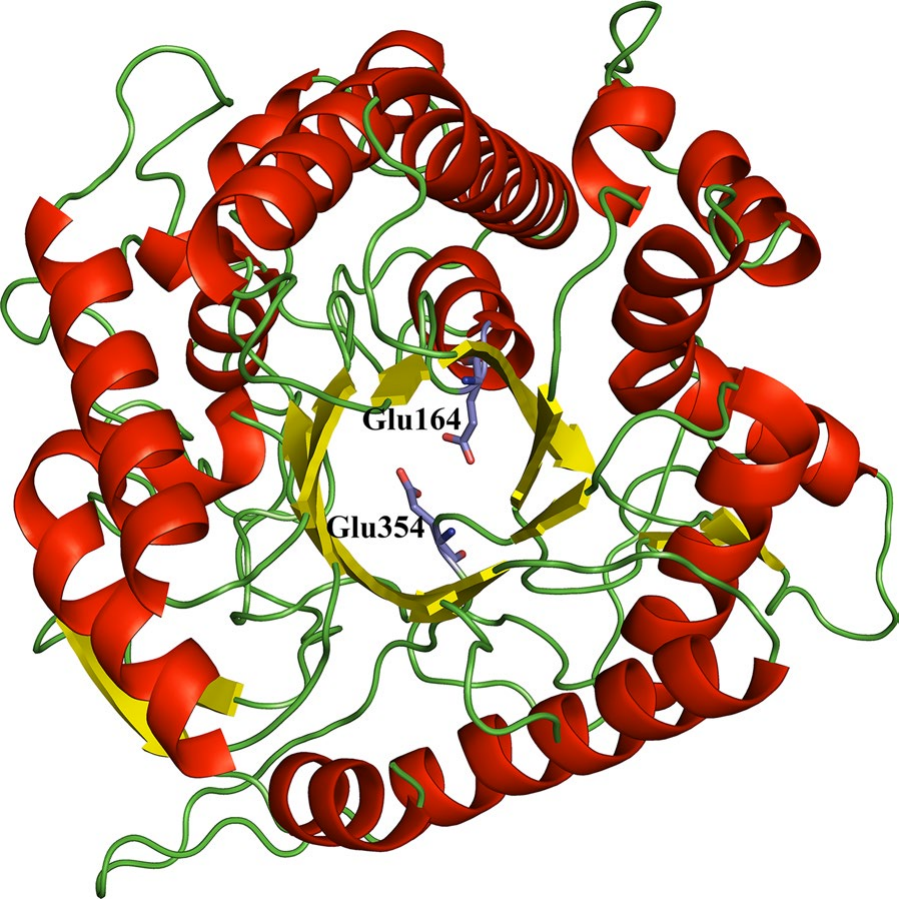
Three-dimensional protein structure of DT-Bgl

The draft genome of *Anoxybacillus* sp. DT3-1 was sequenced as previously reported [19]. An open reading frame for BGL (1362 bp) was identified in the genome sequence of *Anoxybacillus* sp. DT3-1. The gene for DT-Bgl encodes a protein of 453 amino acids, and is expressed intracellularly inside *Anoxybacillus* sp. DT3-1 due to the lack of a signal peptide. Multiple sequence alignments of DT-Bgl with six GH1 BGL enzymes revealed highly conserved sequence elements and its active sites (Fig. 1). DT-Bgl contains the characteristic NEPW (amino acid 163–166) and TENG (353–356) motifs of the GH1 BGL. The two conserved carboxylic acid catalytic residues (Glu164 and Glu354), as shown by highlighted in yellow (Fig. 1), are located at β-strands 4 and 10, and act as the catalytic acid–base and nucleophile, respectively (Fig. 1) [20]. The overall three-dimensional predicted structure of DT-Bgl resembles the typical (β/α)_8_-barrel structure observed in other structurally characterized GH1 family member, with the active site pocket located at the barrel (Fig. 2).

**Fig. 3:**
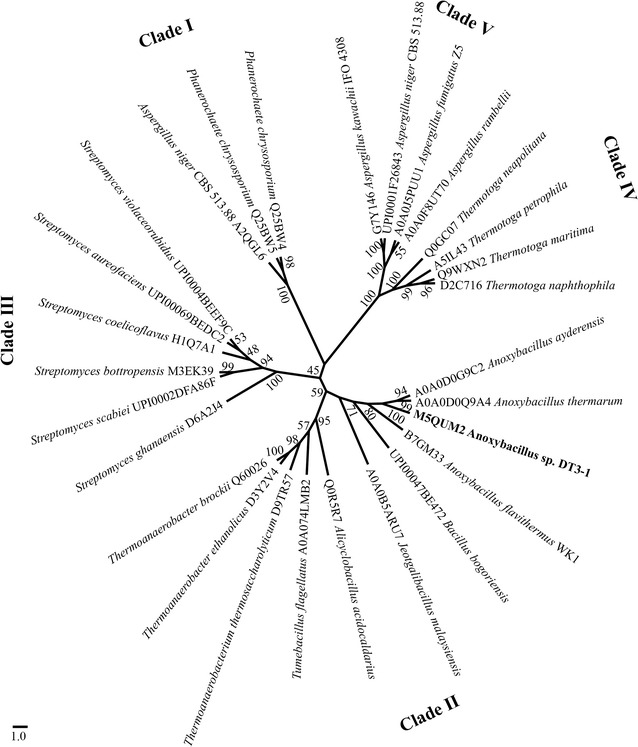
Phylogenetic relationship between DT-Bgl and other β-glucosidases. The numbers associated with the branches refer to bootstrap values (confidence limits), representing the substitution frequencies per amino acid residue. The proteins are identified by their UniProt accession number

The protein sequence showed 92–96 % identity to homologous sequences present in the genomes of *A. ayderensis* [21], *A. thermarum* [22], *A. flavithermus* [23], and other *Anoxybacillus* spp. None of these homologous proteins have been characterized so far. The phylogenetic tree shown in Fig. 3 was constructed by comparing the protein sequence of DT-Bgl with other BGL sequences present in database. β-glucosidases from *Anoxybacillus* spp. clustered together and formed Clade II with BGL from *Jeotgalibacillus malaysiensis*, *Bacillus*, *Tumebacillus*, *Alicyclobacillus*, *Thermoanaerobacterium*, and *Thermoanaerobacter* spp. The similarity between DT-Bgl and the neighboring taxa other than *Anoxybacillus* spp. was in the range of 51–56 %. A previous whole-genome comparison of four *Anoxybacillus* strains and several *Geobacillus* spp. elucidated that proteins from these two genera are closely related [19]. Nevertheless, DT-Bgl exhibited low-protein sequence identity of 42 % to a BGL from *Geobacillus kaustophilus* strain HTA426. Therefore, it is believed that DT-Bgl is a BGL uniquely affiliated to *Anoxybacillus*.

Clade II has been previously described as a group of enzymes constituted of GH1 thermostable β-glucosidases [12]. It is clear from Fig. 3 that Clade II is probably not restricted to enzymes obtained from thermophilic genera, since *Tumebacillus* [24] and *Jeotgalibacillus* [25] are mesophiles. Clade II is phylogenetically closer to Clade III, which is well represented by *Streptomyces* spp. and other mesophilic bacteria, such as *Paenibacillus panacisoli*. Members in Clade I are GH1 β-glucosidases from fungi. Clade IV and Clade V are constituted of enzymes classified in the GH3 family, sourced from fungi and bacteria, respectively.

### Purification of the recombinant DT-Bgl

DT-Bgl was overexpressed in *E. coli* using the expression vector pET28a. Under current experimental conditions, the recombinant DT-Bgl was produced as a soluble protein in large quantity. In contrast to this, some β-glucosidases from other thermophiles, for instance, Tt-BGL from *Thermotoga thermarum* DSM 5069^T^ formed inclusion body in *E. coli* host [26].

**Fig. 4:**
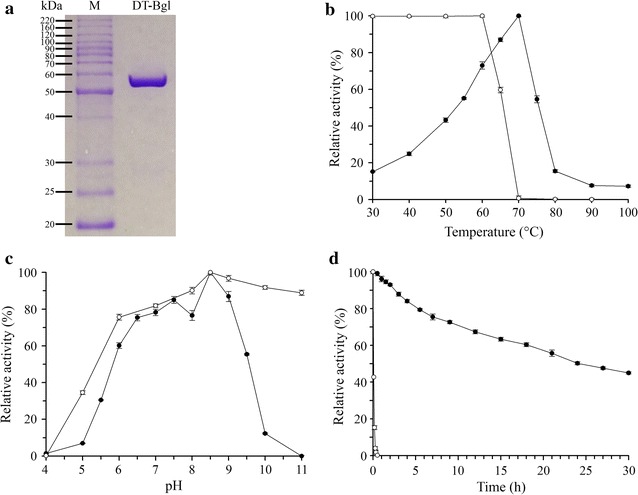
Identification and characterization of DT-Bgl. **a** SDS-PAGE analysis of purified DT-Bgl. M: protein marker with molecular mass in kDa; DT-Bgl: purified DT-Bgl. Effects of temperature (**b**) and pH (**c**) on DT-Bgl activity (-●-) and stability (-○-). **d** Thermostability of DT-Bgl at 60 °C (-●-) and 70 °C (-○-)

The purified fractions of DT-Bgl eluted from an Ni–NTA column were pooled and analyzed by SDS-PAGE. A single band corresponding to a molecular mass of approximately 53 kDa was observed (Fig. 4a) with a specific activity of 1158 U/mg using *p*NPG as the substrate.

### Effects of temperature and pH optimum and stability on DT-Bgl

The biochemical properties of DT-Bgl were determined using the purified recombinant DT-Bgl. When using *p*NPG as substrate, the purified DT-Bgl exhibited maximum activity at 70 °C and pH 8.5, while its relative activity was higher than 70 % of the maximum activity at the temperature range from 60 to 70 °C and pH range from 6.5 to 9.0 (Fig. 4b, c). The optimum temperature of DT-Bgl is comparable or higher than BGLs from *Nasutitermes takasagoensis*, *Thermoanaerobacterium aotearoense*, and *T. thermosaccharolyticum* DSM 571 (Table 1), but DT-Bgl exhibits a much higher optimum pH value. DT-Bgl was stable at pH 6–11, and a negligible reduction in activity was observed when it was incubated at temperatures lower than 60 °C (Fig. 4b, c). The activity half-life of DT-Bgl at 60 °C was 24 h (Fig. 4d). The hydrolysis of cellulose is usually performed under mild conditions at 45–50 °C [27]. Due to the feature of stability at elevated temperature and pH, DT-Bgl is a potential β-glucosidase candidate for wide industrial saccharification processes, including textiles, pulp and paper, and waste treatment for cellulose-degrading function [28].

**Table 1:** Characteristics of DT-Bgl compared with BGL from other organisms

Source	MM (kDa)	T_opt_ (°C)	pH_opt_	Thermostability	*K*_m_^a^ (mM)	*V*_max_ (U^b^/mg)	Reference
*Anoxybacillus* sp. DT3-1	53	70	8.5	60 °C for 24 h^c^	0.22	923.7	This study
*Thermoanaerobacterium aotearoense P8G3#4*	46	60	6.0	50 °C	0.66	180.6	[29]
*Thermoanaerobacterium thermosaccharolyticum* DSM 571	52	70	6.4	60 °C	0.62	64	[12]
*Dictyoglomus thermophilum* DSM 3960	52	90	7.0	90 °C for 5 h^c^	1.15	–	[30]
*Thermotoga thermarum* DSM 5069T	55	90	4.8	90 °C	0.59	142	[26]
*Nasutitermes takasagoensis*	60	65	5.5	60 °C	0.67	8	[31]
*Rhizomucor miehei* NRRL 5282	82	70	5.0	70 °C for 35 min^c^	0.12	468.2	[32]

^a^ Determined by measuring the rate of hydrolysis using *p*NPG as substrate

^b^ One unit of enzyme activity is defined as the amount of enzyme necessary to liberate 1 μmol of *p*-nitrophenol per min under the assay conditions

^c^ Half-life activity of the enzyme

Despite the optimum temperature of DT-Bgl was 70 °C, its stability was drastically reduced over time. DT-Bgl lost its activity completely after pre-incubated in the absence of *p*NPG substrate for 30 min at 70 °C (Fig. 4b, d). This indicated that *p*NPG substrate may stabilize DT-Bgl at high temperatures. Therefore, for subsequent analyses, the reaction was conducted at 60 °C instead of 70 °C, since the enzyme is more stable at 60 °C.

### Effect of metal ions and reagents on purified DT-Bgl

The influence of various metal ions (2 or 5 mM) on DT-Bgl activity was investigated (Table 2). None of the tested metal ions significantly elevated the activity of DT-Bgl, suggesting that the enzyme does not require any of these metals as co-factors. Similar observation was noticed for BGL of *T. thermarum* DSM 5069^T^ [26]. Besides, it has been reported that Ca^2+^ improves the activity of multiple GH enzymes, including BGL from *T. thermarum* DSM 5069^T^ and *T. thermosaccharolyticum* DSM 571 [12, 26]. However, CaCl_2_ did not influence the catalytic efficiency of DT-Bgl. Salts, such as NaCl and KCl, showed little effect on its activity; however, DT-Bgl was sensitive to Fe^3+^, Ni^2+^, Co^2+^, Zn^2+^, and Mn^2+^ ions (Table 2). Nevertheless, Mn^2+^ was known to be an activator for BGL from *T. thermosaccharolyticum* DSM 571 [12]. The addition of 2 or 5 mM urea had no significant effect on its activity. DT-Bgl retained approximately 90 % of its original activity in the same molarity of EDTA indicating that DT-Bgl is not a metalloprotein. Furthermore, DT-Bgl tolerated a variety of chemical reagents, including 1 % (v/v) Tween-20, 1 % (v/v) Tween-40, DMSO, or Triton-100; however, it lost its activity in 5 % (v/v) Tween-20, 5 % (v/v) Tween-40, or 5 % (v/v) Tween-80.

**Table 2:** Effects of metal ions and chemicals on DT-Bgl activity

	Relative activity (%)			Relative activity (%)	
	2 mM	5 mM		1 % (v/v)	5 % (v/v)
Control	100.00 ± 0.01	100.00 ± 0.01	Control	100.00 ± 0.01	100.00 ± 0.01
CaCl_2_	79.33 ± 0.02	70.58 ± 0.01	Tween-20	91.62 ± 0.02	1.61 ± 0.00
NaCl	96.08 ± 0.01	91.13 ± 0.03	Tween-40	106.40 ± 0.01	0.67 ± 0.00
KCl	96.14 ± 0.01	94.19 ± 0.02	Tween-80	61.98 ± 0.01	0.25 ± 0.01
MgCl_2_	84.48 ± 0.01	80.17 ± 0.01	Triton X-100	99.26 ± 0.01	90.01 ± 0.02
FeCl_3_	7.07 ± 0.04	0.03 ± 0.00	DMSO	104.20 ± 0.01	92.29 ± 0.03
NiCl_2_	68.06 ± 0.00	3.56 ± 0.00			
CoCl_2_	87.69 ± 0.00	9.32 ± 0.01			
NH_4_Cl	91.79 ± 0.03	88.18 ± 0.01			
ZnCl_2_	10.21 ± 0.00	2.27 ± 0.00			
MnCl_2_	46.38 ± 0.04	34.81 ± 0.00			
Urea	100.62 ± 0.01	98.59 ± 0.01			
SDS	72.39 ± 0.03	16.70 ± 0.01			
EDTA	90.53 ± 0.02	89.94 ± 0.00			

### Kinetic parameters

**Fig. 5:**
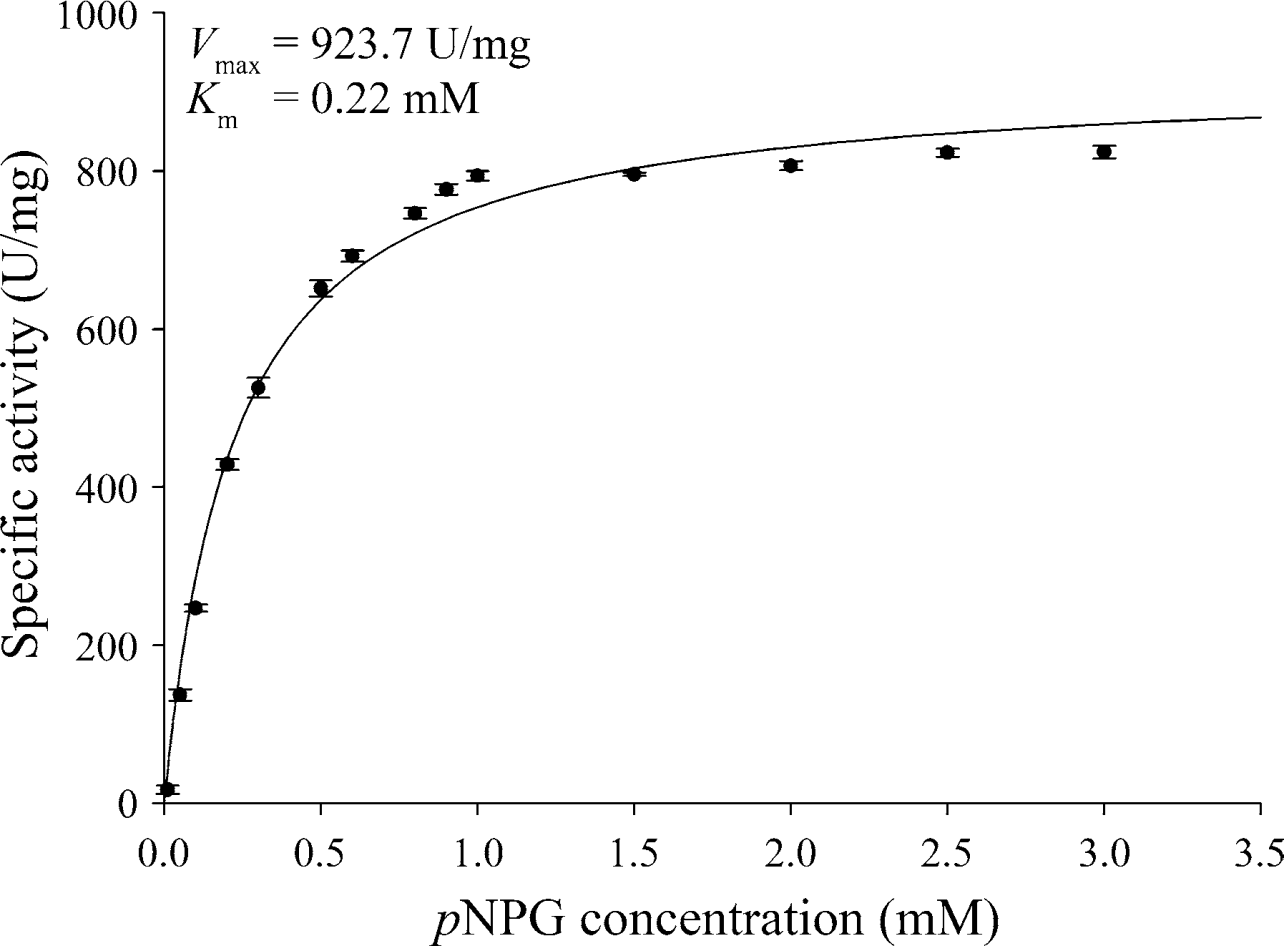
Nonlinear Michaealis–Menten plot of purified DT-Bgl using *p*NPG as substrate

Using *p*NPG as substrate, DT-Bgl showed *K*_m_ value of 0.22 mM, *V*_max_ of 923.7 U/mg, and a *k*_cat_ value of 812.7 s^−1^ using Michaelis–Menten function (Fig. 5). The comparison of these values to closest taxa shown in Fig. 3 could not be performed because of lack of information on these BGLs and the homologues. *K*_m_ of DT-Bgl was found to be lower than the *K*_m_ values reported for the BGLs of other microbial species, including *T. thermosaccharolyticum* and *T. thermarum* (Table 1).

### Substrate specificity

DT-Bgl showed broad substrate-cleaving activity (Table 3). Despite cellobiose is the main substrate for β-glucosidase, DT-Bgl was unable to hydrolyse completely the dimeric compound to glucose monomer using 1 U of purified DT-Bgl at 60 °C for 15 min (Table 3). Using the identical experimental setup, the enzyme showed >90 % substrate depletion activity against sophorose and salicin and completely hydrolysed laminaribiose (Table 3). This emphasizes the importance of DT-Bgl in the hydrolysis of biomass by containing the activities of both laminaribiase and cellobiase. It also showed low hydrolysing activity towards β-gentiobiose (21 %) and lactose (33 %). Negligible hydrolysis was detected when maltose and sucrose were used as substrates. Interestingly, DT-Bgl also showed high hydrolysis efficiencies on cellotriose, cellotetraose, cellopentaose, and cellohexaose (Table 3). These data suggested that DT-Bgl was able to convert these cellodextrins to glucose efficiently, where this finding is in contrast to the hydrolysis preference of BGL obtained from *Aspergillus niger* [33], *Pichia pastoris* [31], and *Trichoderma koningiopasis* [34], where the enzyme efficiency decreased as the length of cello-oligosaccharides increased [31]. It is not clear the maximum length of substrate (degree of polymerisation) for DT-Bgl, since we have not reacted the enzyme with any linear cello-oligosaccharides greater than cellohexaose. Similar to most β-glucosidases, DT-Bgl was not reactive towards complex substrates, including carboxymethylcellulose sodium salt (CMC-Na), Avicel^®^ PH-101, Sigmacell cellulose 101, α-cellulose, and xylan. No sugars were liberated from these complexes even when the reaction time was increased to 24 h. Based on substrates specificity, β-glucosidases are divided into three groups: (i) broad range β-glucosidase, (ii) cellobiase, and (iii) aryl-β-glucosidase. Collectively, according to the hydrolysis ability of DT-Bgl, the enzyme can be assumed to belong to the first group “broad range β-glucosidase”.

**Table 3:** Hydrolysis of various substrates by DT-Bgl

Substrate (concentration, 1 % (w/v)	Linkage of the glycosyl group	Substrate depletion^a^ (%)	Product formation^b^ (µg)		
			Cellobiose	Glucose	Galactose
Cellobiose	β-(1 → 4)Glc	93.61 ± 1.02	–	85.65 ± 0.96	–
Sophorose	β-(1 → 2)Glc	99.93 ± 0.01	–	135.93 ± 0.29	–
Laminaribiose	β-(1 → 3)Glc	100.00 ± 0.00	–	85.20 ± 0.71	–
Lactose	β-(1 → 4)Gal	33.01 ± 5.79	–	37.01 ± 0.04	37.63 ± 0.03
β-gentiobiose	β-(1 → 6)Glc	21.23 ± 1.41	–	37.84 ± 0.02	–
Salicin	β-salicyl alcohol glucoside	93.81 ± 0.35	–	42.51 ± 0.07	–
Sucrose	α-(1 → 2)Fru	0.40 ± 6.66	–	0.00 ± 0.00	–
Maltose	α-(1 → 4)Glc	0.00 ± 0.00	–	0.00 ± 0.00	–
Cellotriose	β-(1 → 4)Glc	100.00 ± 0.00	13.86 ± 0.00	76.11 ± 0.00	–
Cellotetraose	β-(1 → 4)Glc	100.00 ± 0.00	11.82 ± 0.00	70.40 ± 0.00	–
Cellopentaose	β-(1 → 4)Glc	100.00 ± 0.00	14.82 ± 0.00	114.56 ± 0.00	–
Cellohexaose	β-(1 → 4)Glc	100.00 ± 0.00	11.63 ± 0.00	57.94 ± 0.02	–
Xylan	β-(1 → 4)Xyl	0.00 ± 0.00	0.00 ± 0.00	0.00 ± 0.00	–
CMC-Na	β-(1 → 4)Glc	0.00 ± 0.00	0.00 ± 0.00	0.00 ± 0.00	–
Avicel^®^ PH-101	β-(1 → 4)Glc	0.00 ± 0.00	0.00 ± 0.00	0.00 ± 0.00	–
Sigmacell 101	β-(1 → 4)Glc	0.00 ± 0.00	0.00 ± 0.00	0.00 ± 0.00	–
α-Cellulose	β-(1 → 4)Glc	0.00 ± 0.00	0.00 ± 0.00	0.00 ± 0.00	–

^a^Calculated using the following formula: $\frac{{Initialamountofsubstrate{-}Amountofsubstrate}}{Initialamountofsubstrate} \times 100$

^b^The experiment was carried out by reacting 100 μL of substrate with 10 μL of enzyme as described in the “Methods” section

### Effect of glucose on DT-Bgl activity

**Fig. 6:**
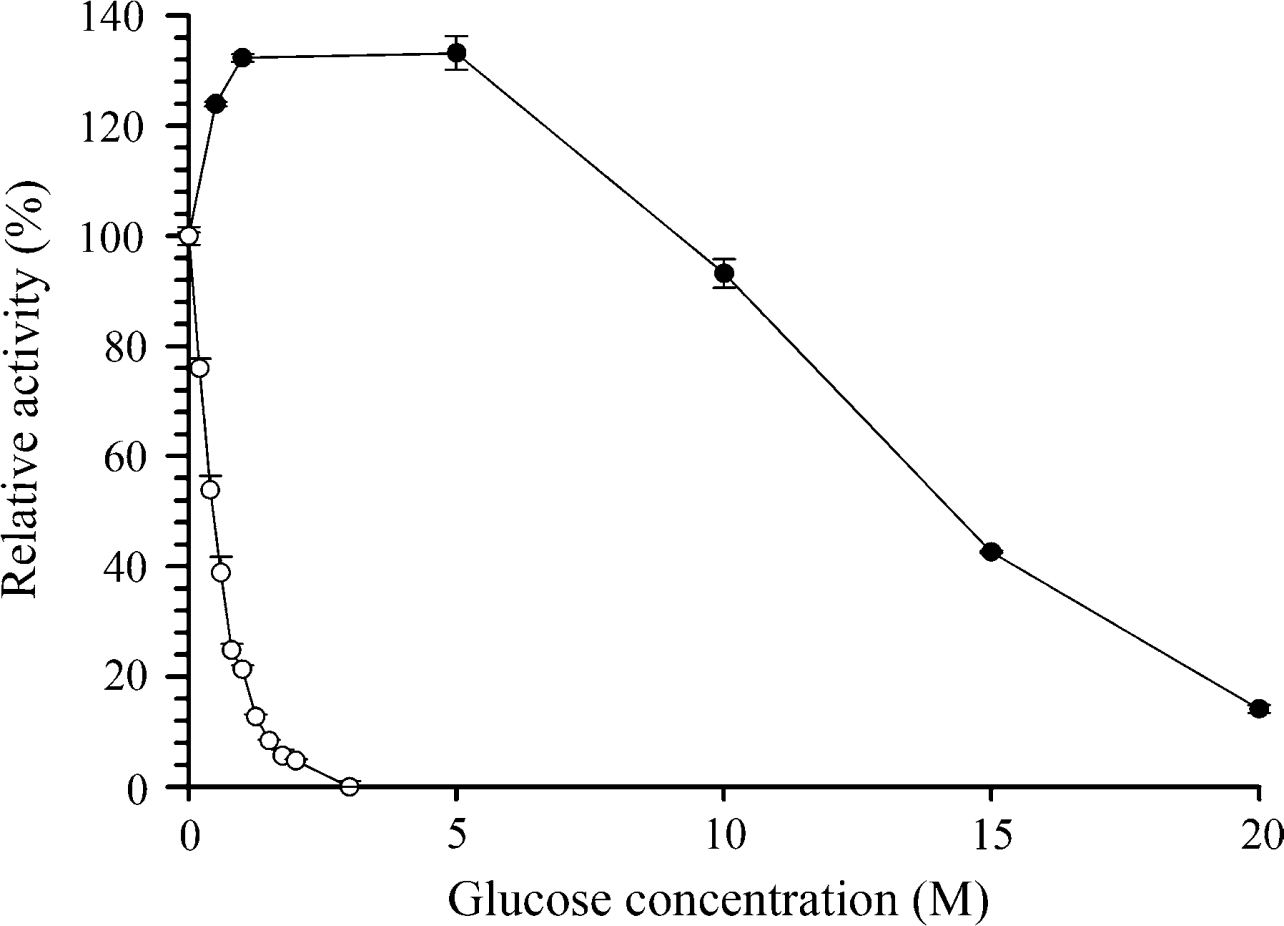
The effects of glucose on DT-Bgl activity towards *p*NPG (-●-) and cellobiose (-○-)

DT-Bgl activity was not affected by glucose up to 5 M when *p*NPG was used as a substrate. The enzyme retained 93  and 43 % relative activity in 10 and 15 M glucose, respectively (Fig. 6). Glucose inhibition in DT-Bgl is lower than β-glucosidase from *T. thermosaccharolyticum* DSM 571, which was known to be one of the glucose-tolerant β-glucosidases [12]. When cellobiose was used as substrate, DT-Bgl retained 76  and 54 % relative activity in 0.2 and 0.4 M glucose, respectively. DT-Bgl was fully inhibited in 3 M glucose (Fig. 6).

## Conclusions

To our knowledge, this is the first study of the heterologous expression of the GH1 β-glucosidase gene from *Anoxybacillus* genus. The DT-Bgl was found to be stable and active in a broad range of pH and temperatures, and it did not require co-factors for activation. DT-Bgl efficiently hydrolyses cellobiose, cellotriose, cellotetraose, cellopentaose, and cellohexaose, and was defined as a “broad range β-glucosidase”. Notably, DT-Bgl has high resistant to glucose up to 5 M. Thus, this study provides a useful novel BGL, which may be used to improve the enzymatic conversion of cellulosic biomass to glucose.

## Methods

### Bioinformatic analyses

Multiple amino acid sequence alignment of DT-Bgl and other selected GH1 BGL enzymes was carried out using the Clustal Omega (Fig. 1). The protein sequences used for the alignment were retrieved from UniProt (http://www.uniprot.org/), with the following accession numbers: DT-Bgl: *Anoxybacillus* sp. DT3-1 (M5QUM2), *Thermoanaerobacter ethanolicus* (D3Y2V4), *Thermoanaerobacterium thermosaccharolyticum* (D9TR57), *Streptomyces scabiei* (UPI0002DFA86F), *Streptomyces coelicoflavus* (H1Q7A1), *Phanerochaete chrysosporium* (Q25BW5), and *Aspergillus niger* CBS 513.88 (A2QGL6). Putative catalytic residues for DT-Bgl were inferred from the sequence alignment. The position of secondary structures of DT-Bgl was predicted with ESPript [35].

Phylogenetic tree among DT-Bgl and other β-glucosidases was constructed using the neighbor-joining method. The results were evaluated with 1000 bootstrap replicates. All the methods above were conducted in the Molecular Evolutionary Genetics Analysis 6.0 software (MEGA, Version 6.0) [36], and the phylogenetic tree was redrawn using FigTree (http://tree.bio.ed.ac.uk/software/figtree/). The tertiary model of DT-Bgl was predicted by CPHmodels 3.2 Server [37], using BGL of halothermophile *Halothermotrix orenii* (PDB id: 3TA9; 54.2 % sequence identity) as template [38].

### Cloning and expression of DT-Bgl

Stock culture of *Anoxybacillus* sp. DT3-1 was grown on solid (15 g/L agar) or liquid media containing 4.0 g/L of peptone, 4.0 g/L of tryptone, 4.0 g/L of yeast extract, 2.0 g/L of NaCl, and 1.0 g/L of MgSO_4_, as reported previously [18]. Genomic DNA was purified using the Wizard Genomic DNA Purification Kit (Promega, Madison, USA). The DT-Bgl ORF was PCR-amplified using F5′-ACG CCC GGA TCC ATG TTT CAG TTT CC-3′) and R5′-CAC CTC GAG TTT GTC GTT GTC TAC TTG C-3′) containing the *Bam*HI and *Xho*I (underlined sequences) restriction sites, respectively. The gene was cloned into pET-28a, and transformed into *Escherichia coli* BL21 (DE3) for its expression. The recombinant *E. coli* was cultured overnight in LB medium supplemented with 50 μg/mL kanamycin at 37 °C with shaking (200 rpm). Two milliliters of culture were inoculated into 200 mL of fresh LB/kanamycin and incubated at 37 °C with shaking at 200 rpm. When OD_600_ reached to 0.6, protein expression was induced by adding filtered sterilised 0.4 mM isopropyl-β-d-thiogalactopyranoside, and the culture was incubated at 37 °C under shaking condition (200 rpm) for 3 h.

### Purification of recombinant DT-Bgl

The induced *E. coli* BL21 (DE3) cells were lysed using the B-PER^®^ Bacterial Protein Extraction Reagent (Thermo Scientific, Waltham, USA). The crude enzyme was dialysed against sodium phosphate buffer (20 mM, pH 7.4) overnight at 4 °C and then loaded onto an Ni–NTA Superflow column (Qiagen, Venlo, Netherlands) using an ÄKTA start system (GE Healthcare, Uppsala, Sweden). The column was equilibrated with binding buffer (20 mM sodium phosphate buffer, pH 7.4, containing 500 mM NaCl and 60 mM imidazole). The resin was washed with binding buffer, and bound protein was eluted with elution buffer (20 mM sodium phosphate buffer, pH 7.4, containing 400 mM NaCl and 350 mM imidazole). The purified enzyme was dialysed against glycine-NaOH buffer (100 mM, pH 8.5) overnight at 4 °C.

### Enzyme assay

β-glucosidase activity was determined by measuring the hydrolysis of *p*NPG (Calbiochem, Darmstadt, Germany). The reaction mixture contained 1.0 mL of 20 mM *p*NPG in 100 mM glycine-NaOH (pH 8.5) and 100 μL DT-Bgl. After incubation at 60 °C for 15 min, the reaction was stopped by adding 1.0 mL ice-cold 1 M Na_2_CO_3_. The released *p*-nitrophenol was measured using a spectrophotometer at 405 nm. The enzyme assays were performed in triplicates. Enzyme- and substrate-free controls were also incubated and measured under the same conditions. One unit (U) of DT-Bgl activity was defined as the amount of enzyme liberating 1 µmol of *p*-nitrophenol per min per mL under the assay conditions.

### Effect of temperature and pH on DT-Bgl

The optimum temperature and pH of DT-Bgl were determined by reacting the enzyme with *p*NPG for 15 min in the ranges of 30–100 °C and pH 4.0–11.0, respectively. The effects of pH on β-glucosidase activity and stability were tested using 100 mM sodium acetate buffer (pH 4.0–5.5), sodium phosphate buffer (pH 6.0–8.0), or glycine-NaOH buffer (pH 8.5–11.0) at 60 °C. The enzyme activity obtained at the optimum temperature or pH was used to calculate the relative percentage of enzyme activity at varying temperature and pH values. The temperature stability was determined by incubating the enzyme for 30 min without *p*NPG at 60 °C and measuring the residual activity.

### Determination of kinetic parameters

The apparent Michaelis–Menten constant (*K*_m_), maximum velocity (*V*_max_), and turnover number (*k*_cat_) for the purified DT-Bgl were assessed by measuring the rate of hydrolysis of *p*NPG at various concentrations (0.01–3.0 mM) at 60 °C for 15 min in glycine-NaOH buffer (100 mM, pH 8.5). The enzymatic kinetic parameters were determined from the Michaelis–Menten function using the SigmaPlot software version 12.0 (Systat Software, Chicago, USA).

### Effect of metal ions and chemical reagents on DT-Bgl

The activity of DT-Bgl was evaluated by reacting the enzyme under standard assay conditions with *p*NPG supplemented with various metal ions (CaCl_2_, NaCl, KCl, MgCl_2_, FeCl_3_, NiCl_2_, CoCl_2_, NH_4_Cl, ZnCl_2,_ and MnCl_2_) or chemical reagents (urea, dimethyl sulfoxide- DMSO, Tween-20, Tween-40, and Tween-80, Triton X-100, sodium dodecyl sulfate, and EDTA) (Table 2).

### Substrates hydrolysis by DT-Bgl

To test the ability of DT-Bgl to degrade various substrates (Table 3), 100 μL 1 % (w/v) of these compounds was reacted with 10 μL 1 U of purified DT-Bgl. All reactions were carried out in glycine-NaOH buffer (100 mM, pH 8.5), at 60 °C for 15 min, unless specified. Reactions were stopped by boiling for 5 min, and the samples were analyzed on the HPLC 1260 system (Agilent Technologies, Santa Clara, USA), using a 7.8 × 300 mm Rezex RPM-Monosaccharide Pb^+2^ column (Phenomenex Inc, Torrance, USA) fitted with a 7.8 × 50 mm LC guard column filled with the same stationary phase. An Agilent 385-Evaporative Light Scattering Detector was used, and the nebulizer and evaporator were kept at 30 °C with a nitrogen flow rate of 1.6 slm (standard liters per min). Nanopure water, filtered through a nylon membrane filter (0.45 µm pore size, Millipore, Darmstadt, Germany) and degassed, was used as the mobile phase. All the insoluble particles were filtered through a polytetrafluoroethylene syringe filter membrane (0.45 µm pore size) prior to injection into the column. All substrates and standards used were of high purity grade.

### Effect of glucose on DT-Bgl

The effect of glucose on purified DT-Bgl was evaluated by reacting *p*NPG or cellobiose in the presence of glucose at various concentrations using the standard assay procedure and HPLC analysis, respectively. The relative activity (%) was defined as the relative value to the activity of a control without glucose.

## Abbreviations

AA: auxiliary activities
BGL: β-glucosidase
CMC-Na: carboxymethylcellulose sodium salt
GH: glycoside hydrolase
HPLC: high-performance liquid chromatography
SDS-PAGE: sodium dodecyl sulfate–polyacrylamide gel electrophoresis
*p*NPG: para-nitrophenol-α-d-glucopyranoside
